# Diagnostic Accuracy and Post-Procedural Complications Associated with Ultrasound-Guided Core Needle Biopsy in the Preoperative Evaluation of Parotid Tumors

**DOI:** 10.1007/s12105-021-01401-w

**Published:** 2021-12-17

**Authors:** Monika Jering, Marcel Mayer, Rubens Thölken, Stefan Schiele, Andrea Maccagno, Johannes Zenk

**Affiliations:** 1 Department of Otolaryngology, Head and Neck Surgery, Medical Faculty, University Hospital Augsburg, University of Augsburg, Sauerbruchstaße 6, 86156 Augsburg, Germany; 2 Department of Otolaryngology, Head and Neck Surgery, Medical Faculty, University of Cologne, Kerpener Straße 62, 50931 Cologne, Germany; 3 Institute of Mathematics, University of Augsburg, Universitätsstraße 2, 86159 Augsburg, Germany; 4 Institute of General Pathology and Molecular Diagnostics, Medical Faculty, University of Augsburg, Stenglinstraße 2, 86156 Augsburg, Germany

**Keywords:** Core needle biopsy, Neoplasm, Parotid gland, Parotid malignancy, Facial nerve

## Abstract

Correct diagnosis of a parotid neoplasm based on histology preoperatively is of utmost importance in order to guide patient management. The aim of this study was to evaluate the diagnostic accuracy of an ultrasound-guided core needle biopsy of a parotid lesion and to describe associated post-procedural complications. A retrospective study was conducted between January 2015 and March 2021 of all patients who were referred to a tertiary care center for evaluation of a parotid lesion and who underwent core needle biopsy due to high-risk features or when malignancy was suspected on clinical examination or ultrasonography. Patient characteristics, histological findings, and post-procedural complications were recorded and evaluated. Among 890 patients referred for evaluation of a parotid lesion, in 138 patients a core needle biopsy was undertaken. On the basis of core needle biopsy findings, 11 lymphomas and 82 non-lymphoma malignancies were diagnosed in the parotid gland. The sensitivity of the core needle biopsy predicting the accurate tumor type was 97.56% (95% CI 91.47–99.70%) and the specificity 94.64% (95% CI 85.13–98.88%). The accuracy for the correct histopathological diagnosis was 93.48% (95% CI 87.98–96.97%). Post-procedural minor complications occurred in 19 patients (13.8%). In conclusion, a core needle biopsy can identify malignancy in the parotid gland with high sensitivity and specificity in a safe manner and therefore guide surgical treatment.

## Introduction

Salivary gland tumors comprise a wide range of different tumor entities of benign and malignant tumor type [1]. A malignant tumor occurs in 20% of all lesions found in the parotid gland [2–4]. Although malignancy is rare in the parotid gland, it is of the utmost importance to identify the tumor type preoperatively in order to guide further treatment. The close relationship of the parotid gland to the facial nerve and the varying surgical approaches can make the treatment of a parotid mass challenging. Repeat surgery of the parotid gland should be avoided due to scarring and therefore this presents a high risk for facial nerve injury.

The physical examination of a patient with a parotid lesion cannot reliably distinguish benign from malignant masses as signs suggestive of malignancy such as facial nerve palsy may be absent [5, 6]. Ultrasound of the salivary gland is routinely used to detect malignancy of the parotid gland and provides the surgeon with valuable information about tumor size, location, and morphology of the lesion [7, 8]. However, ultrasound needs to be performed by a skilled and experienced sonographer, and malignancy cannot be determined in all cases by this non-invasive method [9].

Ultrasound-guided core needle biopsy (CNB) seems to be a method safe to differentiate between a malignant and benign parotid lesion [10–12]. CNB is only performed at a few salivary gland centers. Previous studies have shown that compared with other less invasive methods such as fine-needle aspiration (FNA), CNB has higher sensitivity (0.94) and specificity (0.98) [10, 13]. For FNA, the sensitivity and specificity vary and were reported 70.0–80.0% and 87.5–97.0% in distinguishing between a malignant and benign parotid lesion [1, 14–17]. Establishing an accurate tissue diagnosis preoperatively is essential in guiding operative or non-operative treatment of the parotid tumor and for prognostication [18].

CNB is described to be a relatively safe procedure with a low complication rate and a higher diagnostic yield when compared with FNA [10]. The most commonly reported complication is a hematoma in 0.5% [10, 13], and tumor cell seeding from the biopsy seems to be rare [1, 19].

The aim of this study was to investigate the diagnostic accuracy in predicting malignancy of a CNB and the associated post-procedural complications of this minimally invasive method in patients referred to a university hospital specialized in salivary gland diseases in whom suspicion for malignancy arose based on physical examination or ultrasonographic findings.

## Materials and Methods

In this retrospective study, we included all patients who were referred to the university hospital Augsburg, Germany, between January 2015 and March 2021 for evaluation of a parotid mass and who underwent core needle biopsy. The study was approved by the local ethics committee (2017/20) and all patients included in this study provided written informed consent. All patients underwent a preoperative ultrasound of the affected parotid gland, which was performed by an experienced clinical investigator using Acuson S2000 (Siemens Medical Solution, Erlangen, Germany) and a linear transducer (4.0–9.0 MHz). The size, location, echogenicity, and configuration of the parotid lesion were assessed on ultrasound. All patients who were eligible for a surgical intervention were operated on. A core needle biopsy of the parotid mass was performed if one of the following criteria was met: suspected malignancy on ultrasound, a lesion within the parotid gland along with new occurrence of facial palsy, rapid increase in the size of a parotid lesion, a painful parotid lesion, and known malignancy in the head and neck area. Malignancy on ultrasound was suspected when tumor margins were inhomogeneous, in case of visible necrosis, or lymph node metastases. CNB was performed under ultrasound guidance and at least three samples were obtained with a single-use, automated spring-loaded core needle (14 or 16 Gauge) using sterile technique after administration of local anesthesia and incision of the skin. The tissue was evaluated by paraffin section histology. The histopathologic workup included fixing in buffered 4% formalin, usually overnight. Hematoxylin and eosin (HE) and Alcian-Peroidic acic Schiff (Alcian-PAS) stained slides served as the basis for the histological diagnosis. Dependent on the results of conventional stains evaluation, further staining and advanced tissue-based analyses like immunohistochemistry, fluorescence in situ hybridization, or molecular analyses were performed. Histopathological results of the CNB were interpreted by two board certified pathologists. Patients who did not undergo an ultrasound-guided CNB of the parotid gland (n = 752) were excluded from this study. Data were extracted from the local database. Patient characteristics, histopathological findings, the clinical course, and post-procedural complications were collected and evaluated. All patients with oral anticoagulation were included in this study. A medication with acetylsalicylic acid was not paused, whereas any other anticoagulants were stopped for at least five days.

Descriptive statistical analyses were performed, and the sensitivity and specificity were calculated. Sensitivity and specificity were calculated by 2 × 2 tables, and confidence intervals were analyzed by the Clopper–Pearson interval. Data are presented as mean (standard deviation) or median (interquartile range) for continuous variables, and proportions for categorical variables.

Statistical analyses were performed using SPSS (IBM SPSS Statistics 25.0, IBM, New York City, NW, USA).

## Results

Overall, 890 patients were evaluated for a parotid gland lesion during the course of the study and out of this patient cohort only138 (15.5%) patients underwent CNB owing to concerning features on clinical examination and were thus included in this study. Out of these 138 patients, the majority of patients were male (72.5%), and the mean age of the study population was 76.2 ± 11.8 years for malignant tumors and 64.2 ± 16.6 years for benign lesions (Table 1). On the basis of CNB malignancy was identified in 82 (59.4%) patients and a benign tumor was diagnosed in 56 (40.6%) patients. The most common malignant tumor based on histopathology was a metastasis of a cutaneous squamous cell carcinoma (SCC), which was detected in 44 patients (53.7%). The most commonly diagnosed benign lesion was a Warthin’s tumor (WT) in 30 patients (21.7%).

**Table 1:** Baseline characteristics and post-procedural complications following CNB according to tumor type

	Benign tumor n = 56	Malignant tumor n = 82
Age (years)	64.2 ± 16.6	76.2 ± 11.8
Sex		
Male (n = 100)	40 (40.0%)	60 (60.0%)
Female (n = 38)	16 (42.1%)	22 (57.9%)
Post-procedural complications		
Hematoma (n = 7)	3 (42.9%)	4 (57.1%)
Seroma (n = 4)	4 (100.0%)	0 (0%)
Infection (n = 10)	6 (60.0%)	4 (40.0%)
Facial paralysis (n = 2)	0 (0.0%)	2 (100.0%)

Values are reported as frequency (%) or mean ± standard deviation

Table 1 shows the observed post-procedural complications. The most common complication was a post-procedural local infection, which occurred in ten patients (7.2%). All patients with this complication were treated with an oral antibiotic and the need for surgical intervention did not arise. A post-procedural hematoma occurred in seven patients (5.0%), all of whom were prescribed oral anticoagulants such as acetylsalicylic acid. These hematomas were managed conservatively with the application of a pressure dressing and surgical evacuation was not necessary. In an additional two patients the post-procedural hematoma became infected and was successfully treated with antibiotics. Temporary facial nerve palsy due to local anesthesia, immediately after injection of the anesthetic was recorded as a complication in two patients and spontaneously resolved within two hours of the intervention. No permanent facial palsy occurred.

Among the 82 patients who were diagnosed with a malignant tumor 15 different entities were identified (Table 2). Among the 56 patients who were diagnosed with a benign tumor 12 different benign tumor entities were diagnosed on the basis of CNB (Table 2).

**Table 2:** Histopathological types of tumor type

	Number of patients (n)
Malignant tumors	
Metastasis of squamous cell carcinoma	44
Lymphoma	11
Salivary duct carcinoma	5
Malignant melanoma	4
Adenoid cystic carcinoma	3
Mucoepidermoid carcinoma	3
Merkel cell carcinoma	3
Myoepithelial carcinoma	3
Basal cell adenocarcinoma	2
Metastatic renal cell carcinoma	2
Adenocarcinoma	1
Acinar cell carcinoma	1
Round cell sarcoma	1
Carcinoma ex-pleomorphic adenoma	1
Metastatic breast cancer	1
Benign tumors	
Warthin tumor	30
Pleomorphic adenoma	7
Basal cell adenoma	4
Cyst	3
Chronic sialadenitis	3
Sclerosing tissue	3
Sarcoidosis	1
Lipoma	1
Chondrite lesion	1
Sjögren’s syndrome	1
Granulomatous lymphadenopathy	1
Mononucleosis	1

All tumor dignities are counted by occurrence. Each tumor type was calculated individually

**Fig. 1:**
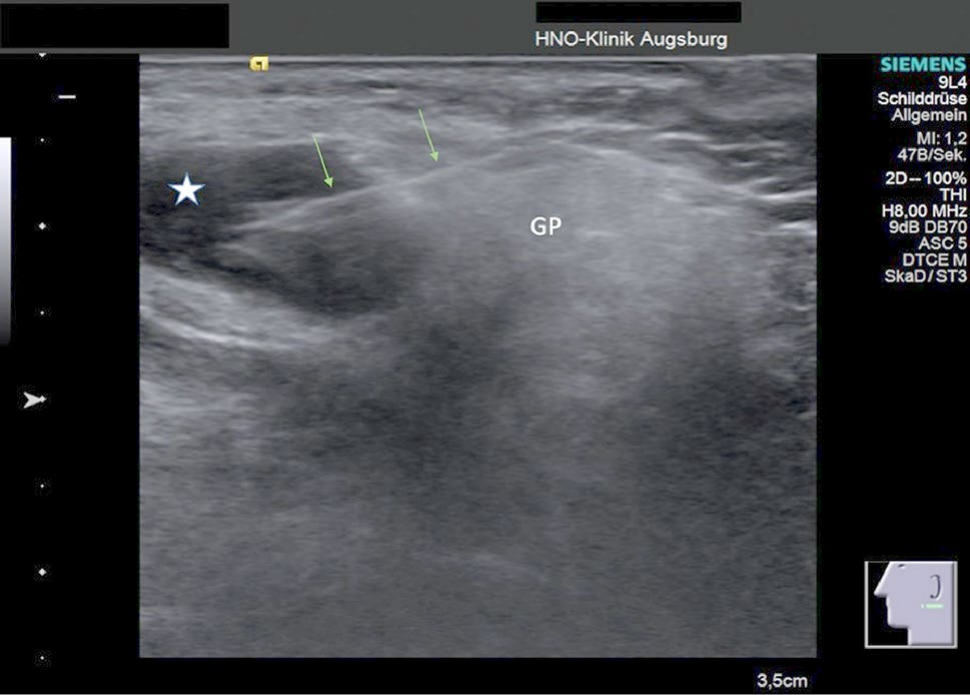
Ultrasound-guided core needle biopsy of a parotid tumor. Representative B-mode ultrasound image of a parotid gland neoplasm (indicated by a star). The arrows point at the core needle (CN). The glandula parotis is marked by *GP*

**Fig. 2:**
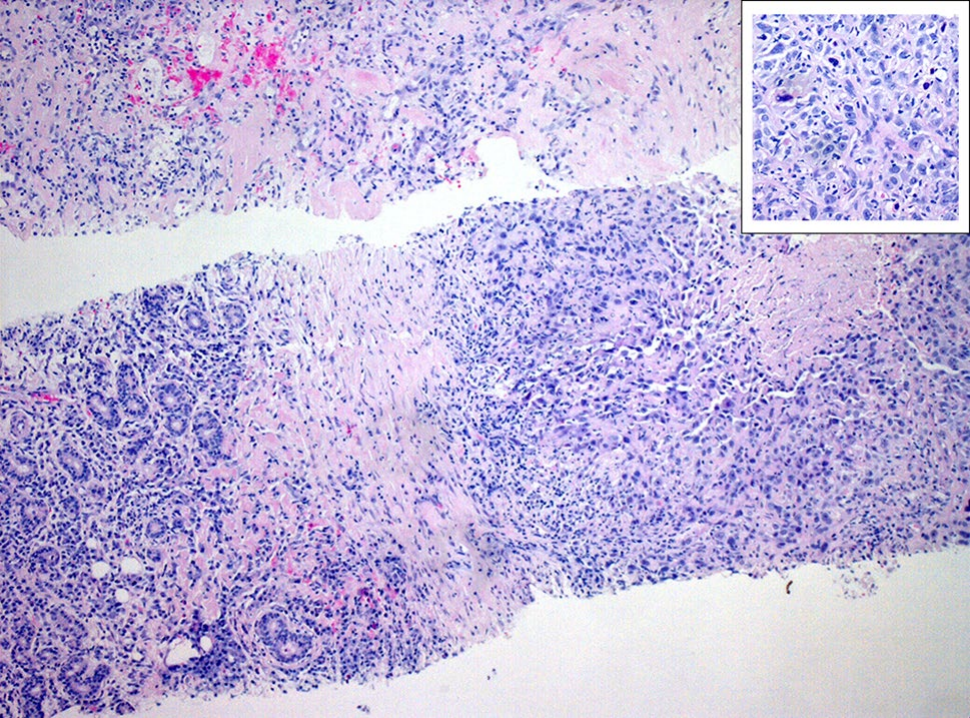
Histopathological results of a core needle biopsy of a squamous cell carcinoma. This histopathological finding (40 × magnification) of a core needle biopsy of a lesion of the parotid gland demonstrates on the left-hand side small amounts of regular salivary tissue, adjacently the poorly differentiated squamous cell carcinoma

**Fig. 3:**
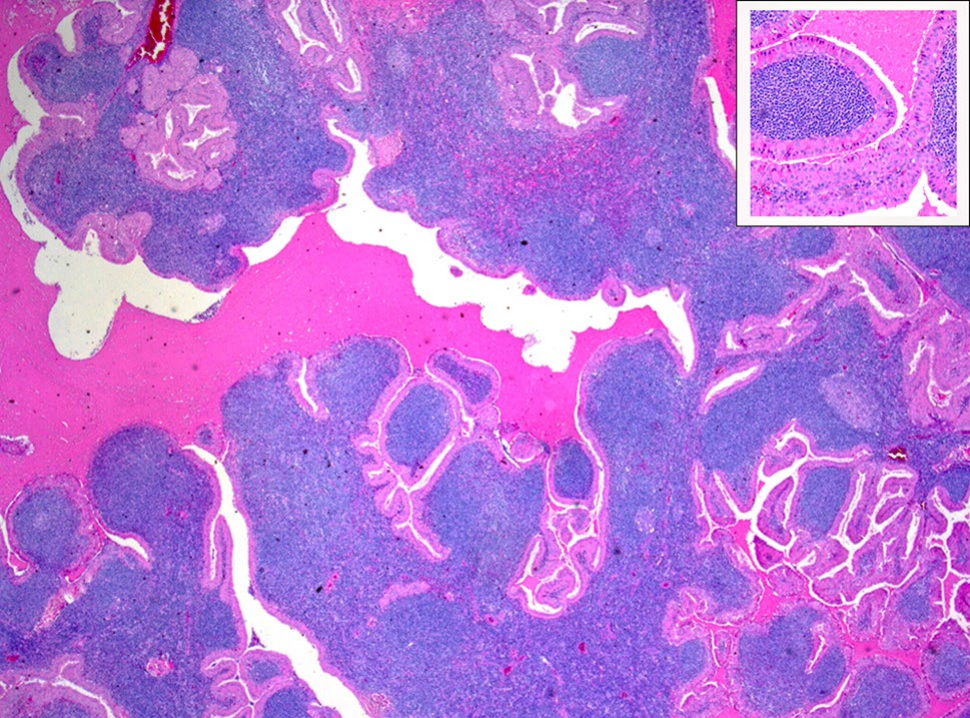
Histopathological results of a core needle biopsy of a Warthin tumor. Figure shows the histopathological findings (12.5 × magnification) of a core needle biopsy of a Warthin tumor. Tissue with double layer of epithelial cells lying on dense lymphoid stroma

In eleven patients a lymphoma was identified on the basis of CNB, thus obviating the need for additional surgical intervention. In all cases the subclassification of the lymphoma was successful. In three patients, two synchronous malignancies were found on CNB, and these were verified postoperatively by the definitive histopathological results from the surgical specimen: two patients with a lymphoma and SCC and one patient with a myoepithelial carcinoma and SCC. The specimen obtained from CNB was insufficient to establish the correct histopathological diagnosis in nine patients (6.0%). Therefore, these patients had to undergo another CNB, open biopsy, or intraoperatively a frozen section evaluation. In these cases, we found insufficient material or necrotic tissue, which could not lead to the correct diagnosis. In five patients (3.6%) a benign tumor could not correctly be specified. In two cases malignancy was not correctly identified and these patients had to undergo an open biopsy, as malignancy was preoperatively suspected. In three cases malignancy could not be excluded by CNB. These patients also underwent an open biopsy (Table 3). In the remaining 124 patients (89.9%) the histopathological results from the CNB were sufficient to yield a diagnosis. Figure 1 shows an example of an ultrasound-guided core needle biopsy. Figures 2 and 3 display histopathological findings of core needle biopsies of a SCC and a WT.

**Table 3:** Accuracy of a core needle biopsy in determining the tumor type

	Malignant tumor	Benign tumor	
Malignant tumor	80	3	83
Benign tumor	2	53	55
Number of patients	82	56	138

This 2 × 2 table demonstrates the predictive ability of a core needle biopsy. In three cases the tumor type could not be determined, and a second biopsy was recommended by the pathologist

The sensitivity of a CNB in this study population predicting the accurate tumor type and correct tumor differentiation was 97.56% (95% CI 91.47–99.70%) and the specificity 94.64% (95% CI 85.13–98.88%). The accuracy of the CNB predicting the correct histopathological diagnosis and tumor type was 93.48% (95% CI 87.98–96.97%).

## Discussion

Due to the rarity of malignant parotid gland tumors and their diversity and morphologic complexity, it is essential to identify the tumor type preoperatively when malignancy is suspected in order to avoid repeat surgery with its associated increased risk of facial nerve palsy [20]. Furthermore, the correct histopathological diagnosis can help guide the timing of the surgical intervention and inform non-surgical management. Additionally, in elderly patients or patients with multiple comorbidities in whom a surgical intervention may carry a high risk a histopathological examination can be useful in order to inform further treatment options. An ultrasound-guided CNB provides parotid tissue to grade the tumor pathology. As the prognosis of parotid malignancy varies by tumor type, establishing the correct diagnosis based on tissue is essential to individualize treatment decisions [21]. In patients with high-grade malignancy a more radical surgery needs to be performed, which carries the risk of facial nerve palsy. Conversely, in patients with low-grade malignancy a more conservative surgical approach can be chosen, and the facial nerve can be preserved, if the nerve is not infiltrated by the tumor [5].

In this study, we showed that ultrasound-guided CNB could determine the tumor type of a parotid neoplasm with a low rate of post-procedural complications and guide further treatment, as the histological architecture can be evaluated from the CNB specimen and subtyping of lymphoma is possible. In the case of a histologically confirmed lymphoma, an unnecessary surgical intervention may be avoided if the diagnosis is established on CNB and the correct treatment may be promptly instituted. In this study, all patients with a diagnosed lymphoma were referred to the oncologist to initiate the correct treatment.

Reported complication rates associated with CNB are low. Kim et al. investigated 1,315 CNB of parotid lesions and showed that tumor cell seeding did not occur in any case [10]. Congruent with these findings, the reported rate of tumor seeding is very low in other studies and fear thereof should therefore not be a barrier to performing a CNB if indicated [22, 23]. Similarly, in our study, tumor cell seeding was not observed after performing a CNB at our institution. This was evaluated by regular follow-up visits.

The relatively high rate of post-procedural hematomas observed in our study might be due to the use of a large-sized needle (14–16 Gauge) and the high prevalence of oral anticoagulant use among the study population. The anticoagulant therapy was continued, and the hematoma was resorbed in all cases. In all cases, symptoms resolved with conservative management and surgical intervention was not necessary, nor did this complication prolong the hospital stay. Permanent facial palsy with nerve damage did not occur in our study, and other studies describe this complication to be extremely rare [24, 25].

The findings of our study should be interpreted within the context of the limitations of a retrospective study conducted at a single tertiary care center. A limitation to this study is the exclusion of a high number of patients due to missing clinical and sonographic signs of malignancy. Another limitation to this study is that low-grade carcinoma could not be identified preoperatively because of missing clinical and sonographic signs of malignancy. Thus, a CNB was not performed and these patients were not included in this retrospective study. Another limitation to this study is the varying follow-up period ranging from two to 74 months. A tumor cell seeding could not be securely excluded. Furthermore, only CNB was performed in this study, and findings were not compared against FNA. Although FNA carries a lower risk of complications, insufficient aspiration of tissue can limit the ability to differentiate between a benign and malignant neoplasm or yield a non-diagnostic sample, as demonstrated in previous studies [24, 26]. In comparison to CNB, FNA has lower sensitivity and specificity in characterizing the parotid gland tumor [1]. In addition, the results of a FNA have to be evaluated by an on-site cytologist, who may not be available in every hospital setting to make an accurate diagnosis [27, 28].

The goal of this study was to investigate the diagnostic accuracy of a CNB in the evaluation of a parotid lesion in patients with high-risk features and to describe the complications after CNB at a large salivary gland center. Additionally, we investigated the accuracy of CNB in predicting the correct histopathological diagnosis. We demonstrated that CNB could correctly determine the histopathological diagnoses in many cases, as the tissue architecture is preserved [11]. Moreover, the number of nondiagnostic specimens was low. This cost-effective intervention is safe with a low rate of serious post-procedural complications and is less invasive than open surgical biopsy comfort. However, a CNB should be performed by an experienced clinician in order to guarantee a high-quality biopsy and thereby facilitate the histopathological examination. Furthermore, patient treatment can be optimized and adjusted preoperatively on the basis of the histologic findings.

## Conclusion

In conclusion, this study demonstrated that a CNB has a high sensitivity and specificity in the diagnosis of salivary gland tumors in a patient population in whom malignancy is suspected. If malignancy is suspected preoperatively, a CNB should be performed in order to obtain a histological specimen. The procedure is simple, safe, and fast. The risk of post-procedural complications is low. Further treatment can be adapted to the patients’ needs and the perioperative risk for facial nerve palsy can be minimized. In the future we plan a prospective study including all patients presenting with a parotid mass and performing a CNB on all patients.

## Data Availability

Data was generated at the University hospital Augsburg, Germany. The data that support the findings of this study are available on request from the corresponding author.
